# CHILDHOOD HEALTH AND COGNITIVE FUNCTION AT MIDLIFE: EVALUATING THE MEDIATING ROLE OF EDUCATION

**DOI:** 10.1093/geroni/igae098.0279

**Published:** 2024-12-31

**Authors:** Nicole Hair

**Affiliations:** University of South Carolina, Columbia, South Carolina, United States

## Abstract

Educational attainment is one of the strongest predictors of dementia among older adults. It is unclear, however, whether this association reflects the benefits of education per se or early life factors that influence educational attainment. We leverage the introduction of the first measles vaccine in 1963, which contributed to a pronounced and sustained reduction in infectious disease morbidity, to evaluate the effects of better overall health in childhood on cognitive function at midlife and, further, to assess the role of educational attainment in the causal pathway from early life health to later life cognition. Our project links early-life contextual data, e.g., state-level socioeconomic, epidemiologic and policy measures, to the Health and Retirement Study, a longitudinal population-based study of individuals over age 50 with validated cognitive measures. Our analytic sample includes older adults in the War Baby (1942-47), Early Baby Boomer (1948-53), Mid Baby Boomer (1954-59), and Late Baby Boomer (1960-65) cohorts who were born and attended school in the United States. Our analytic approach compares outcomes across birth cohorts with more or less exposure to measles vaccination campaigns in the 1960s and 1970s and across states with higher or lower baseline measles incidence. We find that mass vaccination against measles had positive and significant impacts on both educational attainment (e.g., years completed) and cognitive function (e.g., global cognition and episodic memory) at midlife and that education mediated the association between better overall health in childhood and cognitive function later in life.

